# Trapped in harmful work? How psychosocial job exposure, sex, and education shape occupational mobility in Norway

**DOI:** 10.1186/s12889-026-27523-w

**Published:** 2026-05-09

**Authors:** Lasse Holtar

**Affiliations:** Centre for the Study of Professions (SPS), OsloMet - Oslo Metropolitan University, Oslo, Norway

**Keywords:** Psychosocial job exposure, Occupational mobility, Sex, Education, Linear Probability Models

## Abstract

**Background:**

While the health risks of psychosocial job exposure are well established, the extent to which workers reduce exposure by changing occupations remains unclear. This study examines whether Norwegian workers in high-exposure occupations are more likely to change their occupation compared to those in lower-exposure occupations, whether such changes lead to less exposure, and whether these patterns vary by educational attainment and sex.

**Methods:**

Comprehensive Norwegian register data were linked to a validated, sex-specific psychosocial job exposure index covering 322 occupations to track occupational mobility across different exposure levels from 2006 to 2019. Linear probability models were used to estimate the likelihood of mobility from one year to the next and, amongst those who changed, moving to an occupation with lower exposure. To assess whether observed patterns were driven by large, female-dominated professions, further analyses excluding teaching and health care occupations were estimated. All analyses were stratified by sex, and estimates were reported both overall and by educational level.

**Results:**

Workers in high-exposure occupations were less mobile than workers in lower-exposure occupations. However, much of this pattern was largely shaped by the concentration of highly-educated women in teaching and health care. Among those who changed occupations, women were more likely than men to move into occupations with lower exposure, even when excluding teachers and health care workers. Although the differences weakened when teaching and health care occupations were excluded, higher education did not increase the likelihood of moving into lower-exposure occupations for either sex.

**Conclusions:**

Higher psychosocial exposure is associated with lower occupational mobility in the Norwegian workforce. The low mobility observed among highly-educated women in high-exposure occupation is largely shaped by their concentration in teaching and health care. Taken together, the findings suggests that exposure, sex and education interact in ways that may limit movement out of high-exposure work, particularly for certain occupational groups.

**Supplementary Information:**

The online version contains supplementary material available at 10.1186/s12889-026-27523-w.

## Background

Modern work exposes employees to psychosocial stressors that pose significant risks to their health [1]. The extent of this exposure is often shaped by the interaction between job demands (e.g., workload, time pressure) and job control (e.g., decision-making autonomy) [2]. High-strain jobs, characterized by high demands and low control, are particularly harmful, increasing the likelihood of burnout, mental health disorders, cardiovascular disease, sick leave, and early retirement [1, 3, 4]. However, while the consequences of adverse working conditions are well-documented, far less is known about how workers actively respond to these challenges. This study explores occupational mobility as a potential response, examining whether psychosocial job exposure increases the likelihood of Norwegian workers leaving their occupation in order to alleviate strain.

In discussions about career decisions, particularly those involving remaining in a workplace or organization, it is often implicit that individuals are responsible for their own choices and will leave a workplace when the job is no longer perceived as fulfilling [5–7]. This perspective is grounded in the notion that people act purposively and in accordance with their intentions, carefully weighing the potential rewards of mobility against the associated risks [8, 9]. On the one hand, the rewards of occupational mobility may include reduced exposure to harmful job characteristics, improved working conditions, and better health.

On the other hand, workers must also account for the risks of mobility, such as the potential loss of income, diminished returns on human-capital investments, or uncertainty about whether the new role will effectively reduce harmful exposure. When workers perceive that the expected future rewards of a new role, such as increased control and reduced demands, outweigh the risks of leaving their current role, they are more likely to pursue mobility [8–10]. Because prolonged job exposure can increase dissatisfaction, alternative roles with better working conditions or reduced exposure become especially appealing to workers in high-exposure occupations.

However, even when workers are motivated to leave, not everyone can choose where they work or change occupation at will. For many, barriers such as a lack of educational attainment or occupational segregation can constrain their ability to pursue mobility by defining what is feasible and shaping the opportunities available to them [5]. Although the specialized skills and knowledge that often accompany higher education may draw workers to specific occupations, individuals with higher educational attainment are generally expected to have broader career opportunities [11, 12]. This is because higher education enhances employability and labor market attractiveness [8, 12], and is frequently used by organizations as a key criterion in hiring decisions [13]. Highly educated workers also report greater confidence in finding new opportunities, likely due to increased cognitive abilities and transferable meta-skills [14–16]. In contrast, lower educated workers often face limited opportunities in the labor market due to a lack of qualifications and skills [12].

Similarly, women face distinct challenges to mobility. In the gender-segregated Norwegian labor market, women are disproportionally concentrated in a narrow range of high-strain occupations with limited opportunities for advancement, which substantially limits the set of viable career options available to them [4, 17–19]. Men, on the other hand, are more likely to consider job changes, change jobs more frequently, and experience greater upward labor mobility. Women’s mobility is further constrained by caregiving responsibilities, temporary employment, weaker professional networks, and lower labor market visibility [17, 20, 21].

While these dynamics provide insight into some of the factors driving mobility, significant gaps remain in our understanding of how psychosocial exposure shape workers’ decisions to leave their occupations. Existing research suggests that increased psychosocial job exposure can predict mobility, but most studies focus on turnover intentions rather than actual mobility [22–24]. Studies that do examine actual mobility are typically limited to specific occupational groups [25, 26], and even when workers leave, it remains unknown whether mobility effectively reduces exposure. Furthermore, many studies overlook the previously mentioned barriers that can restrict mobility opportunities for certain groups of workers, such as women and lower educated workers [27, 28].

Accordingly, this study aims to address these limitations by investigating whether Norwegian workers with higher psychosocial exposure levels are more likely to change occupations, whether they transition to occupations with lower levels of exposure, and how these relationships vary with education and sex. To achieve this, the study utilizes comprehensive Norwegian register data covering the entire Norwegian workforce from 2006 to 2019, measuring actual occupational mobility to better understand how workers respond to psychosocial job exposure.

## Method

### Data and variables

The analysis is based on Le, Hermansen and Dahl’s [4] validated Job Strain Index, designed for Norwegian registry-based research. This index addresses gaps in Norwegian working condition data by utilizing gender-specific job exposure matrices (JEMs) constructed from five nationwide work environment surveys in 2006, 2009, 2013, 2016, and 2019 developed by Statistics Norway (SSB). Covering 322 occupations, the JEM is combined with individual-level data from national administrative registers provided by SSB. These registers, linked via the national individual identification number, offer annual information on employment, education, demographics, and other background characteristics for the entire Norwegian workforce.

To ensure consistency, the sample is restricted to individuals with valid four-digit occupational codes included in the JEM. The analysis focuses on individuals who remain employed in consecutive years, as the measurement of occupational mobility requires year-to-year transitions between occupations. Individuals are not required to have continuous employment throughout the entire sample period, but they must be employed in consecutive years to be included.^1^ Because the unit of analysis is the person-year, individuals may contribute multiple observations across the study period, with each year of consecutive employment from *t-1* to *t* constituting a separate observation. The sample is further limited to individuals aged 30–59, as mobility among younger workers may reflect educational choices, early career exploration, or temporary contracts, whereas older workers may be influenced by retirement-related transitions [17]. This yielded a sample of 2,280,874 individuals with at least one period of consecutive annual employment from 2006 to 2019.

Two outcome measures are of interest. First, to assess the extent to which individuals move within the labor market, occupational mobility is used. Every occupation is defined by a unique set of role requirements, including the tasks to be performed and the capabilities needed to succeed. Occupational mobility, therefore, involves transitions that demand new skills, routines, and work environments, often requiring additional training or vocational preparation [12, 29]. Since this study focuses on levels of exposure within specific occupations, the analysis emphasizes occupational mobility rather than job mobility.

The mobility variable is a binary indicator that measures whether an individual changes occupations between consecutive years. An occupational change is defined as a transition to a different four-digit Norwegian standard STYRK-98 occupational code compared to the previous year. The STYRK-98 classification system, based on the International Standard Classification of Occupations (ISCO-88), provides a standardized framework for categorizing occupations in Norway. Using four-digit codes ensures that the analysis captures broader occupational transitions, as individuals must move further away from their initial occupation than if six-digit codes were used. Although this approach captures fewer transitions per year, it avoids counting mobility between closely related roles that are likely to share similar levels of job exposure.^2^

Secondly, to examine whether individuals transition to occupations with lower levels of exposure, a measure of exposure-reducing mobility between consecutive years is used. This is a binary measure that indicates whether an individual transitions to an occupation with an equal or higher exposure level (coded as 0) or to an occupation with a lower exposure level (coded as 1). While the first outcome measure includes all individuals, this measure only includes individuals with an actual occupation change from one year to the next. This separates overall occupational mobility from mobility that specifically involves a transition to occupations with lower psychosocial exposure.

The main independent variable is psychosocial exposure level the year prior to occupational mobility, based on the beforementioned Job Strain Index (Le et al., 2023). The index, rooted in Karasek’s Job-Demands Control Model [2], combines job demands and job control to capture gender-specific psychosocial exposure in different occupations. It is constructed using self-reported data collected by Statistics Norway, with different items designed to capture different aspects of psychosocial exposure. Each item is based on a survey question where respondents were asked to rate their experiences on a scale, indicating the degree to which they experienced a particular demand or level of control.

Job demands were measured using four items: (1) quantitative demands, (2) conflicting ways of doing things, (3) insufficient resources, and (4) contradictory requests. Job control, on the other hand, was measured using six items: (1) decide how to go about the work, (2) decide the pace of work, (3) make important decisions, (4) use skills, (5) develop skills, and (6) monotonous work [4]. Each item was dichotomized by splitting the responses at the median to classify individuals as either exposed or not exposed to that specific item.

To aggregate exposure by occupation, the mean proportion of exposed individuals within each occupation was calculated separately for the four items measuring job demands and the six items measuring job control. These were subsequently averaged to create a single, continuous measure of psychosocial exposure for each occupation and gender, ranging from 0 (no exposure) to 100 (universal exposure). Higher values on the index represent occupations with higher levels of job demands and lower levels of job control, while lower values represent occupations with lower levels of job demands and higher levels of job control [4].^3^

**Fig. 1:**
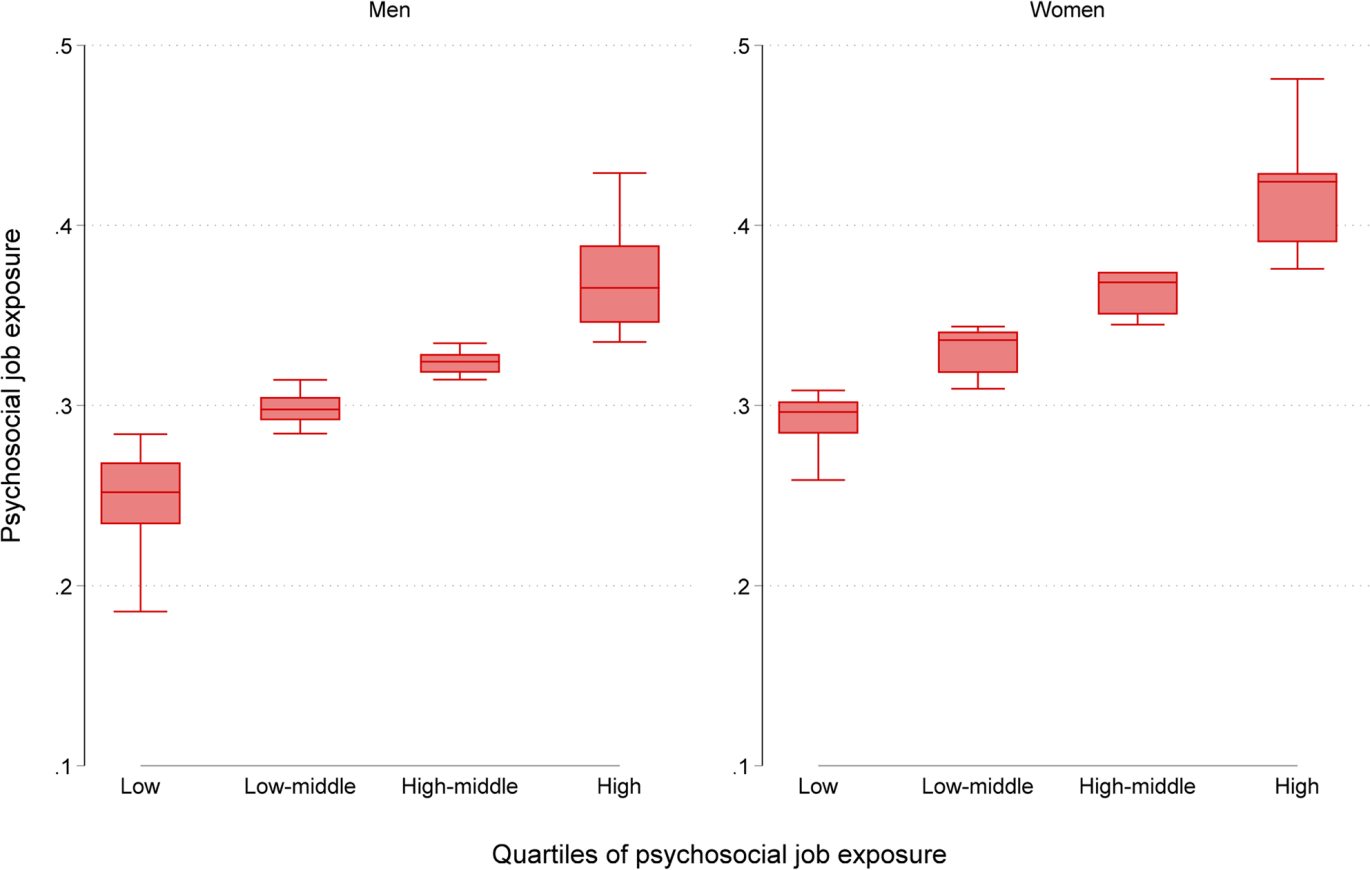
Distribution of psychosocial job exposure, by sex and exposure level. Note: Box-plot displaying the distribution of the continuous psychosocial job exposure index (range 0–100) across quartile-based exposure categories, shown separately for men and women.

Building on the original measure of exposure, the index is further divided into quartiles, creating four different levels of psychosocial exposures for each sex. These levels, illustrated in Fig. 1, are categorized as: (1) Low exposure, (2) Low-middle, (3) High-middle, and (4) High exposure. Because the index is constructed separately for men and women, the same four-digit occupation may fall into different exposure quartiles for each sex. Thus, an occupation may be classified as high-middle exposure among men but high exposure among women. Unlike the traditional classification in Karasek’s Job-Demands Control Model (e.g., low-strain, passive, active, high strain) the present categorization does not cross-classify demands and control into four distinct quadrants. Instead, it represents a gradient of overall psychosocial strain within occupations, where higher quartiles indicate occupations characterized by both higher demands and lower control.

Educational level is operationalized using the highest achieved educational level in the year prior to occupational mobility. Educational level is classified according to the Norwegian Standard Classification of Education (NUS2000), a 6-digit code system that classifies educational activities by level and field, where the first digit represents the educational level. The variable is categorized into four groups: (1) Primary education, which includes pre-primary, primary, and lower secondary education; (2) Secondary education, which combines upper secondary and post-secondary education; (3) Bachelor-level higher education; and (4) Master’s/Ph.d.-level higher education.

To account for observable characteristics that may be associated with both psychosocial job exposure and occupational mobility, the analyses adjust for a range of sociodemographic and employment-related variables. These include immigration background, part-time employment (0 = full-time, 1 = part-time), occupational tenure (continuous years in occupation), marital status (0 = not married, 1 = married), children under 18 (0 = no, 1 = yes), and a quadratic term for age. Previous research has shown that these factors are related to both occupational sorting and mobility [20, 23, 30–32]. In addition, the analyses include long-term sick leave (0 = no, 1 = yes), defined as having at least one sickness-related absence spell exceeding 16 days during the calendar year. This variable captures prior health-related work absence that may influence subsequent mobility decisions.

Table 1 provides a descriptive overview of all the independent variables included in the analyses, presented as either unadjusted percentages or mean values of occupational mobility over the entire study period (2006–2019). The data are disaggregated by sex, allowing for a comparison of occupational mobility patterns between men and women across different levels of the independent variables. All time-varying independent variables are lagged by one year (*t-1*) in order to ensure temporal precedence.

**Table 1:** Descriptives. Percentage of occupational mobility (%), by sex

	Men			Women		
	Mobility	95% CI	Total	Mobility	95% CI	Total
Exposure level						
Low	11,0	10,9–11,0	2,257,368	10,4	10,4–10,5	2,293,645
Low-middle	11,8	11,8–11,9	2,316,414	12,3	12,2–12,3	2,066,321
High-middle	12,5	12,5–12,6	2,082,698	9,8	9,7–9,8	1,827,534
High	10,1	10,1–10,2	2,198,148	7,3	7,3–7,3	2,036,027
Educational level						
Primary educ.	10,6	10,6–10,7	2,148,561	8,6	8,5–8,6	1,975,652
Secondary educ.	11,1	11,0–11,1	4,099,243	10,1	10,0–10,1	2,686,017
BA	12,3	12,3–12,4	1,488,988	10,1	10,0–10,1	2,619,632
MA/Ph.d.	12,5	12,5–12.6	1,117,836	12,3	12,2–12,4	942,226
Immigration back						
Majority	11,2	11,2–11,2	7,458,932	9,8	9,8–9,8	7,058,273
Immigrant	12,3	12,3–12,4	1,075,095	10,8	10,7–10,8	874,041
Second gen.	12,4	12,3–12,5	320,601	11,4	11,2–11,5	291,213
Marital status						
Not married	11,9	11,9–12,0	3,815,877	10,8	10,8–10,9	3,102,161
Married	10,9	10,9–11,0	5,038,751	9,4	9,4–9,5	5,121,366
Children						
No children	10,5	10,5–10,6	3,780,495	8,7	8,7–8,7	3,523,379
Children	12,0	11,9–12,0	5,074,133	10,9	10,9–10,9	4,700,148
Employment type						
Full-time	11,2	11,1–11,2	7,279,355	10,4	10,3–10,4	4,275,724
Part-time	12,3	12,3–12,4	1,575,273	9,5	9,5–9,6	3,947,803
Sick leave						
No sick leave	11,2	11,2–11,2	7,613,178	9,9	9,9–10,0	6,057,995
Sick leave	12,2	12,2–12,3	1,241,450	10,1	10,1–10,1	2,165,532
Tenure (mean)	3,4	3,4–3,4	8,854,628	3,1	3,1–3,1	8,223,527
Age (mean)	41,5	41,4–41,5	8,854,628	41,2	41,2–41,3	8,223,527

The CIs are computed for proportions and reported in percentage points. Given the large sample sizes, intervals are narrow and may appear identical after rounding

*CI *confidence intervals

### Statistical analysis

To investigate the relationship between job exposure and mobility, and how this relationship varies with sex and education, linear probability models (LPM) are employed. This means that estimates can be interpreted directly as differences in probabilities, allowing for easy comparisons across groups and models.^4^ Given the gender-specific psychosocial exposure measure, separate regressions are conducted for men and women for both outcomes of interest. Two models are estimated for each sex. Model 1 includes an interaction between job exposure and education, while model 2 adds all additional covariates. Calendar year dummies are included in all regressions to account for time trends, external shocks, and changes in the distribution of occupations over the sample period. Regression coefficients and their standard errors are presented in Appendix Tables 2 and 3.^5^

To account for the disproportionate concentration of women in high-exposure occupations [18], analyses excluding teachers and health care workers from the sample were also conducted. These occupations exhibit the highest average levels of psychosocial job exposure (see Appendix Fig. 6) and account for approximately 68% of all eligible female person-year observations in the highest exposure category. Workers in these occupations are also generally highly educated, as practicing the profession often requires specialized training and knowledge [35, 36]. Thus, excluding these occupations makes it possible to assess whether any potential findings are driven by large, female-dominated professions where workers are both highly exposed and highly educated. The restricted models are estimated using the same specifications as the main analyses.

 All findings are presented in figures that illustrate estimated predicted probabilities to make the results more accessible and intuitive for readers. First, the probabilities were calculated for each level of exposure (baseline) to provide an overall view of how exposure influences occupational mobility. Then, the probabilities were examined for each combination of exposure and education to explore how the relationship between exposure and mobility varies with education. These probabilities represent the likelihood of the outcome occurring for specific values of exposure and education, averaged across the observed values of the additional independent variables in the data.

## Results

**Fig. 2:**
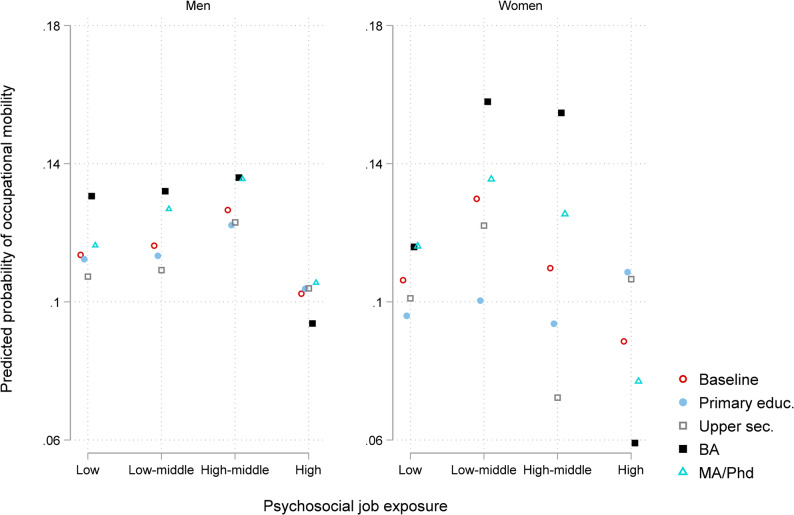
Predicted probability of occupational mobility, by sex and education. Note. Predicted probabilities from linear probability models estimated separately by sex and stratified by education. Models adjust for age, immigration background, part-time employment, occupational tenure, marital status, children, and long-term sick leave. Given the large sample size, CIs are not included as the small intervals make symbols difficult to distinguish

Figure 2 illustrates the predicted probability of occupational mobility by sex and educational level. Among men, mobility probabilities remained relatively stable across exposure levels, ranging from 12.7% to 10.2%, suggesting that exposure level had a limited impact on their likelihood of mobility. Men in high-middle exposure occupations were the most mobile, while those in high-exposure occupations were the least mobile. For women, however, mobility probabilities showed a more pronounced decline in high-exposure occupations. While mobility increased slightly from low to low-middle exposure occupations, it dropped steadily from a peak of 13% in low-middle exposure to a low of 8.9% in high-exposure occupations. Notably, men were more mobile across all exposure levels, except in low-middle exposure occupations, where women were the most mobile.

Examining occupational mobility by education reveals distinct patterns for men and women. Among men, mobility probabilities remained relatively stable across all exposure levels, with little variation by education. Men with higher education were slightly more mobile in low-exposure occupations. At higher exposure, however, men with a bachelor’s degree were somewhat less mobile than their less-educated counterparts, while men with a master’s/PhD exhibited similar mobility levels to those with lower levels of education. Nevertheless, both groups remained close to the baseline probability of 10.2%. Women, on the other hand, exhibited significantly more variation by education. Women with a bachelor’s or master’s/PhD degree were more likely to change occupations in low-middle and high-middle exposure categories compared to women with primary or upper secondary education. In high-exposure occupations, however, this pattern reversed. Women with a bachelor’s or master’s/PhD degree had mobility probabilities of only 5.9% and 7.7%, respectively - several percentage points lower than women without higher education.

**Fig. 3:**
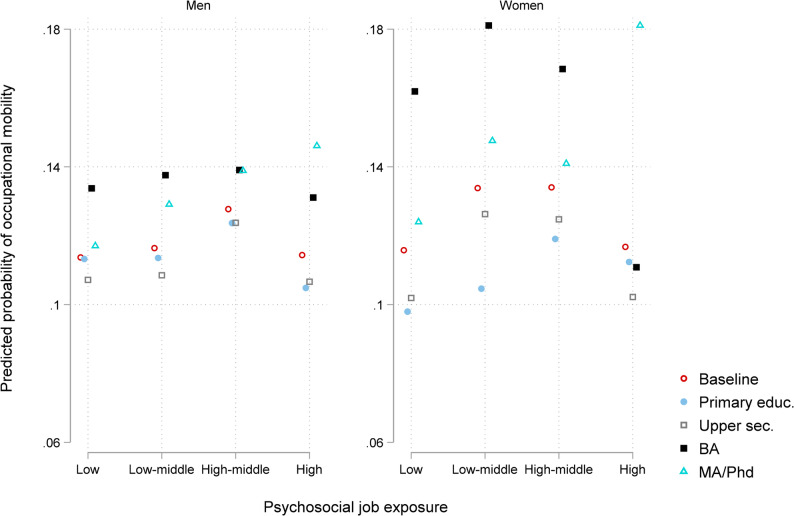
Predicted probability of occupational mobility, excluding health care workers and teachers. Note. Predicted probabilities from linear probability models estimated separately by sex and stratified by education. Models adjust for age, immigration background, part-time employment, occupational tenure, marital status, children, and long-term sick leave. Given the large sample size, CIs are not included as the small intervals make symbols difficult to distinguish

Figure 3 portrays the predicted probability of occupational mobility, excluding health care workers and teachers. For men, the baseline exposure gradient remains broadly similar to the full sample. However, education-specific probabilities within the highest exposure level shift. Mobility among men with a bachelor’s degree increased from 9.4% in the full sample to 13.1% in the restricted sample, while mobility among men with a master’s or PhD degree increased from 10.6% 14.6%. For women, the effects are substantially more pronounced, and the overall negative association between high exposure and occupational mobility is markedly reduced. Most notably, the higher education penalty for highly-exposed women in the full sample largely disappears. Mobility among women with a bachelor’s degree increased to 11.1%, while mobility among women with a master’s/PhD degree increased from 7.7% 18.1%.

**Fig. 4:**
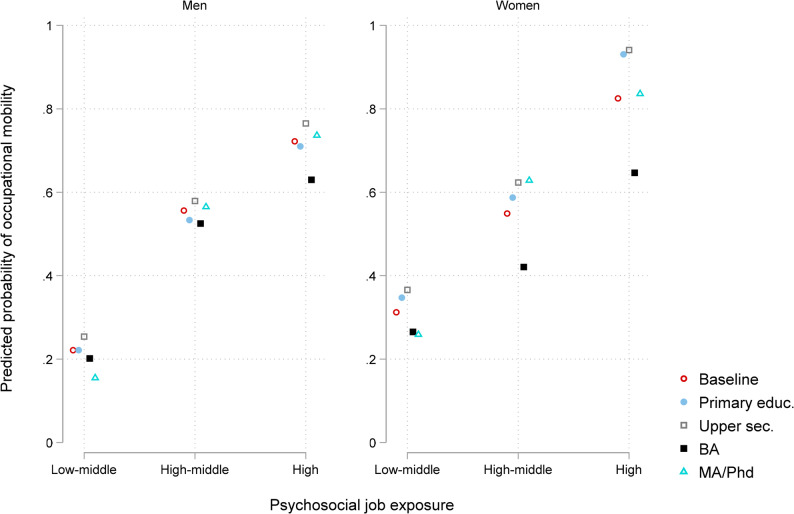
Predicted probability of exposure-reducing mobility, by sex and education. Note. Predicted probabilities from linear probability models estimated separately by sex and stratified by education. Models adjust for age, immigration background, part-time employment, occupational tenure, marital status, children, and long-term sick leave. Given the large sample size, CIs are not included as the small intervals make symbols difficult to distinguish

Figure 4 illustrates the predicted probability of reducing exposure for individuals who changed occupations, separated by sex and educational level. Since opportunities for upward mobility are limited for individuals in high-exposure occupations, their probabilities of exposure-reducing mobility were naturally higher than those in lower-exposure occupations. Therefore, the primary focus is on how these probabilities vary by sex and educational level. For men, the probability of reducing exposure ranged from 24.1% at low-middle exposure to 70.2% at high exposure. At high-middle exposure, the probability was 55.6%, nearly identical to that of women at the same exposure level (54.8%). For women, probabilities were higher than for men overall, ranging from 33.3% at low-middle exposure to 81.5% at high exposure.

As with general mobility, the effect of education on exposure-reducing mobility varied by sex. Educational level had a limited effect on men, with one notable exception: highly exposed men with bachelor’s degrees had a 13.5-percentage-point lower probability of mobility compared to men with upper secondary education. For women, however, education had a much stronger effect. In high-middle exposure occupations, probabilities ranged from a low of 42.1% for women with bachelor’s degrees to a high of 62.9% for those with a master’s or PhD degree. In high-exposure occupations, women with primary or secondary education had probabilities of 91.3% and 92.3%, respectively, compared to 62.8% for women with bachelor’s degrees and 81.7% for those with a master’s or PhD degree.

**Fig. 5:**
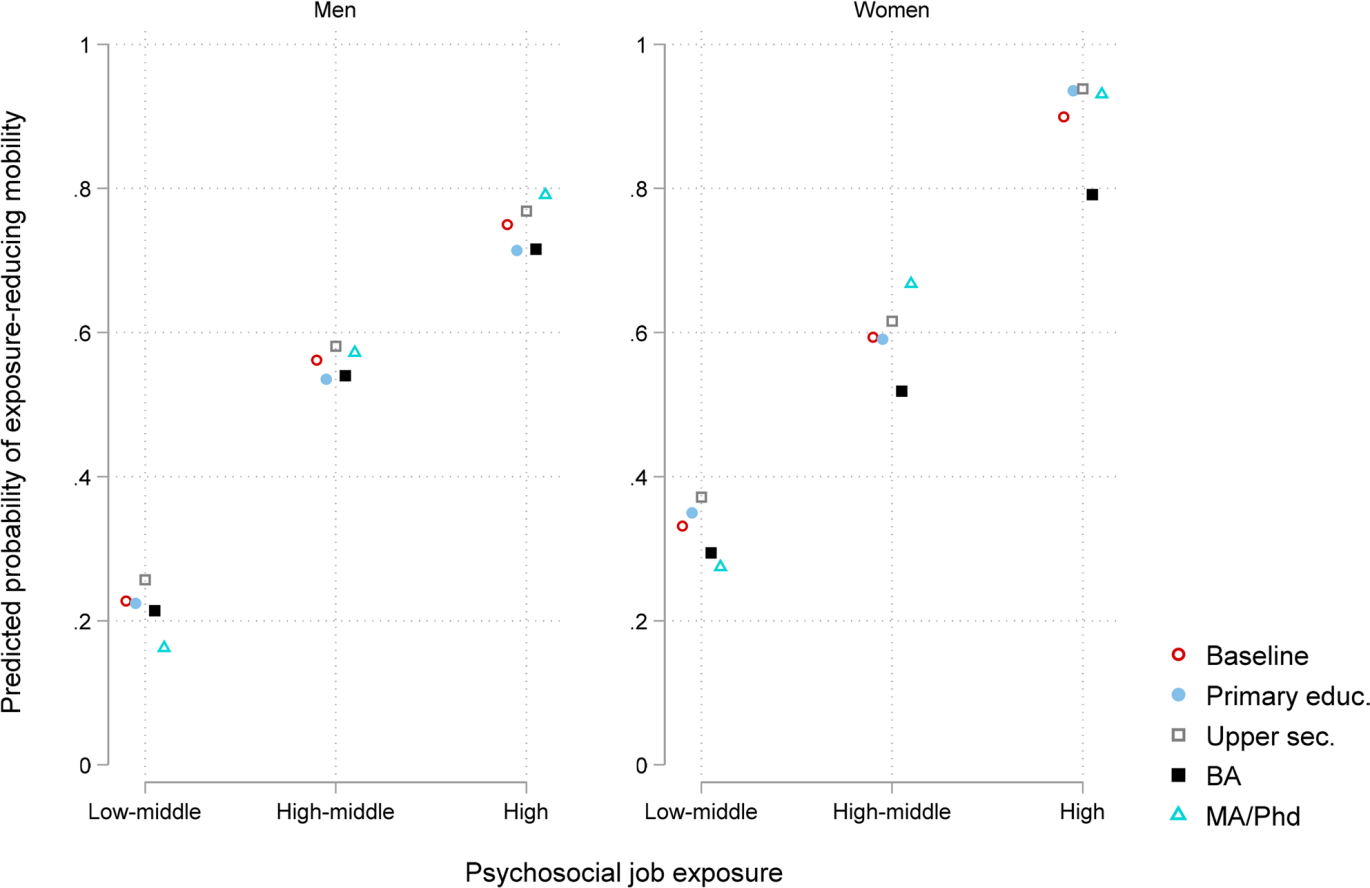
Predicted probability of exposure-reducing mobility, excluding health care workers and teachers. Note. Predicted probabilities from linear probability models estimated separately by sex and stratified by education. Models adjust for age, immigration background, part-time employment, occupational tenure, marital status, children, and long-term sick leave. Given the large sample size, CIs are not included as the small intervals make symbols difficult to distinguish

Figure 5 presents the predicted probability of exposure-reducing mobility, excluding teachers and health care workers. The overall exposure-reducing mobility gradient remained similar to the full sample, but the gender difference in exposure-reducing mobility for highly-exposed workers increased. For men, the baseline probability of exposure-reducing mobility for highly-exposed workers increased from 72.2% to 74.5%, while it increased from 82.5% to 90.0% for women. Education-specific probabilities within the highest exposure category also shift. For men, exposure-reducing mobility for those with a bachelor’s degree increased from 63.0% to 71.6%, and from 73.6% to 79.1% for those master’s or PhD degree. Among women, mobility for those with a bachelor’s degree increased from 64.7% to 79.1%, and for those with a master’s or PhD degree it increased from 83.6% to 93.1%.

## Discussion

These findings challenge the assumption that adverse working conditions drive occupational mobility. Instead, this study found a significant association between higher exposure and decreased occupational mobility. While both men and women in highly exposed occupations exhibited lower mobility compared to lesser-exposed workers, women in these roles were notably less mobile than their male counterparts. Contrary to expectations, higher education did not facilitate greater mobility for either sex in high-exposure occupations. In fact, highly educated women in these roles exhibited a significantly lower probability of mobility compared to their less-educated counterparts, suggesting that higher education does not translate into greater occupational flexibility for highly-exposed workers.

However, excluding teaching and health care occupations indicate that both the pronounced gender difference and negative education gradient are largely shaped by the concentration of highly exposed women within these professions. When these occupations are removed from the analysis, the overall exposure gradient for women is attenuated and the strong education penalty in the highest exposure category disappears. This implies that the lower mobility observed among women in high-exposure occupations reflects characteristics of large, highly-exposed female-dominated occupations rather than a general pattern across the Norwegian labor market.

One such characteristic is the extensive skills and knowledge required to practice many of these occupations [35, 36]. This represent a substantial investment in occupation-specific human capital, which means that transitioning to a different occupation may entail forfeiting the returns on that investment [12, 29, 37]. Higher education may therefore reinforce attachment to a specific professional field rather than expand opportunities for mobility away from it [11, 36]. Relatedly, the specialized nature of many of these professions may significantly narrow opportunities in the labor market. This lack of transferable skills can increase the risks associated with leaving, as workers in these roles face greater uncertainty about finding better opportunities elsewhere, along with the potential loss of income and job security [5, 12].

As a result, these workers may be less inclined to pursue mobility, even when dissatisfaction with working conditions is high [27, 38]. This is particularly true for women, who remain concentrated in a relatively narrow range of occupations with fewer occupational alternatives to choose from [5, 19]. At the same time, many of these workers likely feel a strong commitment to the work they do, as health care workers and teachers are frequently associated with strong professional identities and perceptions of meaningful or socially valuable work. Furthermore, women selecting into these professions may simply emphasize the fulfilling and altruistic part of the job more than economic rewards or career development [39–41].

Although highly exposed women were less likely to change occupation overall, a different pattern emerges when examining exposure-reducing mobility. In fact, when women in high-exposure occupations do transition to other occupations, they are more likely to transition to roles with lower exposure than men. The analysis excluding teaching and health care occupations indicate that this pattern remains largely unchanged, suggesting that women’s tendency towards exposure-reducing mobility is not primarily driven by the concentration of women in these occupations. This may reflect broader gender differences in how straining work is perceived and managed. Women may prioritize reducing exposure as part of balancing work with family-related obligations, as they are more likely to take on caregiving responsibilities [20, 42]. Men, on the other hand, may place less emphasis on reducing exposure, as they tend to downplay or dismiss symptoms and health concerns [43, 44]. High exposure may also be seen as an acceptable trade-off for financial stability or future career advancement, leading men to prioritize roles with greater career potential, even if these roles involve similar or higher levels of exposure [27, 45].

Higher education did not increase the probability of exposure-reducing mobility for highly exposed workers of either sex. Highly educated women, particularly those with bachelor’s degrees, were significantly less likely to transition to lower-exposure occupations compared to their lower-educated counterparts. A similar pattern was observed among men with bachelor’s degrees, who were also less likely to transition to lower-exposure roles. The analysis excluding teaching and health care occupations indicate that this education gradient is partly shaped by the concentration of highly educated workers within these professions, as the negative association between higher education and exposure-reducing mobility is attenuated in the restricted sample. Since these workers have stronger occupational boundaries, their career trajectories may be more organized within the same field or organization [46], even if it involves comparable levels of exposure. Nevertheless, women with bachelor’s degrees continue to exhibit a significantly lower probability of transitioning to lower exposure in the restricted analysis, suggesting that higher education may constrain exposure-reducing mobility for women even beyond health care and teaching professions.

## Limitations

A key limitation of this study is the inability to assess underlying reasons for occupational changes. The data do not distinguish voluntary from involuntary transitions, nor do they indicate whether changes were driven by personal or career-related motives. Although involuntary employment termination is relatively uncommon in Norway due to strong employment protection legislation [47], changes may still reflect motivations unrelated to psychosocial job exposure. This limitation may have obscured the extent to which exposure influences occupational mobility.

A second limitation concerns the operationalization of occupational mobility as transitions between four-digit occupational codes. This approach captures movement across formal occupational categories, but not changes in employer, sector, or work setting within the same occupation. If such within-occupation transitions involve meaningful reductions in psychosocial job exposure, the analysis may underestimate the extent to which workers adjust their exposure without changing occupation.

Similarly, exposure values reflect occupational averages rather than individual-level working conditions. Substantial heterogeneity may exist within occupations, including differences across sectors, contract types, organizational contexts, and individual work arrangements [48]. As a result, some degree of exposure misclassification is possible, and analyses based on individual-level self-reported exposure may yield different patterns.

## Conclusion

Unlike previous research suggesting that psychosocial job exposure leads to increased mobility, this study finds that highly exposed Norwegian workers are more likely to remain in their occupations than those in lower-exposure roles. Women appear particularly affected, as they are less likely than men to change occupations when faced with adverse working conditions. Nevertheless, when women do change occupation, they are more likely than men to move into roles with lower exposure. Surprisingly, higher education did not increase general mobility or the likelihood of moving to occupations with lower exposure for either sex. However, as the analyses excluding teachers and health care workers illustrated, much of the observed effect is shaped by the concentration of highly-exposed workers in large, female-dominated professions. When these professions are excluded, the pronounced gender difference and strong education penalty are substantially reduced, and highly-educated workers exhibit mobility levels comparable to, or higher than, their lower-educated counterparts.

Taken together, these findings indicate that the relationship between exposure, sex and education is conditioned by occupational context. While higher education generally increases employability and labor market attractiveness [12], it may also reinforce attachment and limit mobility opportunities in highly-exposed professions with strong occupational boundaries [11]. Although a strong commitment to meaningful work may further sustain attachment to these professions, workers who remain in these roles are at a greater occupational health risk compared to those who transition out of high exposure [27, 35, 38]. This has serious implications not only for the workers, who face heightened risks to their health, but also for their employers, who, despite retaining much of their workforce, could experience reduced productivity, increased sick-leave and higher rates of early retirement. This highlights the need for broader workplace interventions to reduce harmful exposure, particularly in large, female-dominated professions where opportunities for occupational mobility are low.

## Supplementary Information

Supplementary Material 1.

## Data Availability

The data that support the findings of this study are available from Statistics Norway, but restrictions apply to the availability of these data, which were used under license for the current study, and so are not publicly available.

## Footnotes

1 Approximately 95% of all eligible observations satisfy this requirement, with minimal variation across subgroups.

2 Nurses are a good example of this. In Norway, there are 30 different occupational codes at the seven-digit level for various types of nurses. By collapsing these into four-digit codes, they are grouped into a single occupational category. As a result, a nurse must transition entirely out of the nursing profession for the change to be classified as occupational mobility.

3 See Fig. 6 in the Appendix for an overview of psychosocial exposure by occupational group and sex.

4 While it is a common belief that binary dependent variables require logistic regression or other non-linear models, several scholars [33, 34] argue that LPM is a valid and often advantageous alternative. Nevertheless, logistic regressions are included in the Appendix, Tables 4 and 5, as sensitivity analyses. The results are consistent with those of the original analyses.

5 As psychosocial job exposure may take time to manifest itself and influence mobility decisions, sensitivity analyses were conducted on separate samples of workers with a minimum continuous occupational tenure of 2 and 5 years. The results, presented in Appendix Figs. 7, 8, 9 and 10, are largely consistent with those of the original analyses.

## Abbreviations

JEM: Job exposure matrix
SSB: Statistics Norway
STYRK-98: Norwegian standard for occupational classification used from 1998
LPM: Linear probability models
CI: Confidence interval
