# Supplementary material for: Trapped in harmful work? How psychosocial job exposure, sex, and education shape occupational mobility in Norway
Source: BMC Public Health. 2026 May 9;26:2012. doi: 10.1186/s12889-026-27523-w (PMC13325791; doi:10.1186/s12889-026-27523-w)
Supplement: Supplementary file 1 — Supplementary Material 1. [file 12889_2026_27523_MOESM1_ESM.docx]

**Appendix**

**Figure 6. Average psychosocial job exposure, by occupational group and sex**

Note. Dot plot displaying the mean psychosocial job exposure index (range 0–100) by two-digit occupational group and sex. The index is based on occupational-level averages of job demands and job control, where higher values indicate higher demands and lower control. Exposure is measured using a sex-specific job exposure matrix (JEM).

**Figure 7. Predicted probability of occupational mobility, minimum 2-year tenure**

Note. Predicted probabilities from linear probability models estimated separately by sex and stratified by education. Models adjust for age, immigration background, part-time employment, occupational tenure, marital status, children, and long-term sick leave. Given the very large sample size, CIs are not included as the small intervals make symbols difficult to distinguish.

**Figure 8. Predicted probability of occupational mobility, minimum 5-year tenure**

Note. Predicted probabilities from linear probability models estimated separately by sex and stratified by education. Models adjust for age, immigration background, part-time employment, occupational tenure, marital status, children, and long-term sick leave. Given the very large sample size, CIs are not included as the small intervals make symbols difficult to distinguish.

**Figure 9. Predicted probability of exposure-reducing mobility, minimum 2-year tenure**

Note. Predicted probabilities from linear probability models estimated separately by sex and stratified by education. Models adjust for age, immigration background, part-time employment, occupational tenure, marital status, children, and long-term sick leave. Given the very large sample size, CIs are not included as the small intervals make symbols difficult to distinguish.

**Figure 10. Predicted probability of exposure-reducing mobility, minimum 5-year tenure**

Note. Predicted probabilities from linear probability models estimated separately by sex and stratified by education. Models adjust for age, immigration background, part-time employment, occupational tenure, marital status, children, and long-term sick leave. Given the very large sample size, CIs are not included as the small intervals make symbols difficult to distinguish.

**Table 2. Results from GLS regression of occupational mobility, by sex**

|  | Men | | Women | |
| --- | --- | --- | --- | --- |
|  | (1) | (2) | (1) | (2) |
| *Job exposure* |  |  |  |  |
| Low-middle | 0.001*  (0.001) | 0.001*  (0.001)  0.009***  (0.001)  -0.009***  (0.001)  -0.005***  (0.001)  0.018***  (0.001)  0.004**  (0.001)  0.001  (0.001)  0.000  (0.001)  0.010***  (0.001)  0.006***  (0.001)  -0.005***  (0.001)  0.009***  (0.001)  0.005***  (0.001)  -0.028***  (0.001)  -0.002*  (0.001) | 0.008***  (0.001)  0.001*  (0.001)  0.011***  (0.001)  0.017***  (0.001)  0.043***  (0.001)  0.045***  (0.001)  0.015***  (0.001)  0.032***  (0.001)  0.011***  (0.001)  -0.028***  (0.001)  0.034***  (0.001)  0.007***  (0.001)  -0.003**  (0.001)  -0.074***  (0.001)  -0.050***  (0.001) | 0.006***  (0.001)  -0.004***  (0.001)  0.012***  (0.001)  0.005***  (0.001)  0.020***  (0.001)  0.020***  (0.001)  0.017***  (0.001)  0.038***  (0.001)  0.015***  (0.001)  -0.026***  (0.001)  0.041***  (0.001)  0.011***  (0.001)  -0.007***  0.001)  -0.070***  (0.001)  -0.052***  (0.001) |
| High-middle | 0.020***  (0.001) |  |  |  |
| High | 0.002**  (0.001) |  |  |  |
| *Educ. level* |  |  |  |  |
| Secondary educ. | 0.004***  (0.001) |  |  |  |
| BA | 0.030***  (0.001) |  |  |  |
| MA/Ph.d. | 0.017***  (0.001) |  |  |  |
| *Job exposure x educ. level* |  |  |  |  |
| Low-middle x upper sec. | -0.001  (0.001) |  |  |  |
| Low-middle x BA | 0.003**  (0.001) |  |  |  |
| Low-middle x MA/Ph.d. | 0.009***  (0.001) |  |  |  |
| High-middle x upper sec. | 0.002**  (0.001)  -0.008***  (0.001)  0.007***  (0.001)  0.001  (0.001)  0.032***  (0.001)  -0.008***  (0.001) |  |  |  |
| High-middle x BA |  |  |  |  |
| High-middle x MA/Ph.d. |  |  |  |  |
| High x upper sec. |  |  |  |  |
| High x BA |  |  |  |  |
| High x MA/Ph.d. |  |  |  |  |
| *Part-time* |  | 0.019***  (0.000)  -0.004***  (0.000)  -0.000***  (0.000)  0.008***  (0.000)  0.003***  (0.000)  -0.005***  (0.001)  0.003**  (0.001)  -0.001  (0.001)  0.005***  (0.002)  -0.003***  (0.001)  0.001  (0.002)  0.009***  (0.001)  0.004**  (0.002)  0.003***  (0.001)  0.016***  (0.000) |  | 0.014***  (0.000)  -0.004***  (0.001)  -0.000***  (0.000)  -0.004***  (0.000)  -0.006***  (0.000)  0.006***  (0.001)  0.008***  (0.001)  -0.003***  (0.001)  0.000  (0.002)  -0.013***  (0.001)  0.002  (0.002)  0.011***  (0.001)  -0.004**  (0.002)  0.007***  (0.001)  0.006***  (0.000) |
| *Tenure* |  |  |  |  |
| *Age^2^* |  |  |  |  |
| *Married* |  |  |  |  |
| *Children* |  |  |  |  |
| *Married x children* |  |  |  |  |
|  |  |  |  |  |
| *Immigration status* |  |  |  |  |
| Descendent |  |  |  |  |
| Immigrant |  |  |  |  |
| *Job exposure x immigration status* |  |  |  |  |
| Low-middle x descendent |  |  |  |  |
| Low-middle. x immigrant |  |  |  |  |
| High-middle x descendant  High-middle x immigrant  High x descendant  High x immigrant  *Sick leave* |  |  |  |  |
| Constant | 0.814***  (0.058) | -3.837***  (0.068) | 2.933***  (0.058) | -3.280***  (0.070) |
| *N*  *R^2^* | 8,854,629  0.002 | 8,854,628  0.012 | 8,223,527  0.009 | 8,223,527  0.022 |
| *Note*. Reported standard errors (in parentheses) clustered on individuals.  Reference categories: low exposure and primary education.  Calendar year dummy variables included in regressions.  Signiﬁcance levels: ***.01; **.05; *.1 | | | | |

|  | Men | | Women | |
| --- | --- | --- | --- | --- |
|  | (1) | (2) | (1) | (2) |
| *Job exposure* |  |  |  |  |
| High-middle | 0.302***  (0.003) | 0.315***  (0.003)  0.493***  (0.003)  0.033***  (0.003)  -0.020***  (0.003)  -0.067***  (0.003)  0.013***  (0.003)  0.012***  (0.004)  0.098***  (0.004)  0.022***  (0.004)  -0.060***  (0.004)  0.093***  (0.005) | 0.217***  (0.003)  0.576***  (0.004)  0.036***  (0.003)  -0.054***  (0.003)  -0.057***  (0.003)  0.017***  (0.004)  -0.077***  (0.004)  0.149***  (0.005)  -0.017***  (0.005)  -0.218***  (0.005)  -0.012**  (0.006) | 0.241***  (0.003)  0.578***  (0.004)  0.019***  (0.002)  -0.082***  (0.003)  -0.088***  (0.003)  0.017***  (0.004)  -0.085***  (0.004)  0.130***  (0.005)  -0.008*  (0.005)  -0.202*** (0.005) -0.006 (0.006) |
| High | 0.461***  (0.002) |  |  |  |
| *Educ. level* |  |  |  |  |
| Secondary educ. | 0.041***  (0.002) |  |  |  |
| BA | -0.013***  (0.003) |  |  |  |
| MA/Ph.d. | -0.064***  (0.003) |  |  |  |
| *Job exposure x educ. level* |  |  |  |  |
| High-middle x upper sec. | 0.014***  (0.003)  0.024***  (0.004)  0.105***  (0.004)  0.027***  (0.004)  -0.050***  (0.004)  0.106***  (0.005) |  |  |  |
| High-middle x BA |  |  |  |  |
| High-middle x MA/Ph.d. |  |  |  |  |
| High x upper sec. |  |  |  |  |
| High x BA |  |  |  |  |
| High x MA/Ph.d. |  |  |  |  |
| *Part-time* |  | -0.083***  (0.001)  -0.000  (0.000)  0.000  (0.000)  0.034***  (0.002)  0.038***  (0.001)  -0.016***  (0.002)  -0.001  (0.005)  -0.075***  (0.003)  -0.028***  (0.006)  -0.015***  (0.004)  -0.012***  (0.007)  -0.029***  (0.004)  -0.020***  (0.002) |  | -0.099***  (0.001)  -0.003***  (0.000)  -0.000***  (0.000)  0.023***  (0.002)  0.021***  (0.002)  -0.004  (0.003)  -0.031***  (0.005)  -0.054***  (0.003)  0.040***  (0.007)  -0.024***  (0.004)  0.040***  (0.008)  0.032***  (0.005)  -0.017***  (0.001) |
| *Tenure* |  |  |  |  |
| *Age^2^* |  |  |  |  |
| *Married* |  |  |  |  |
| *Children* |  |  |  |  |
| *Married x children* |  |  |  |  |
|  |  |  |  |  |
| *Immigration status* |  |  |  |  |
| Descendent |  |  |  |  |
| Immigrant |  |  |  |  |
| *Job exposure x immigration status* |  |  |  |  |
| High-middle x descendent |  |  |  |  |
| High-middle x immigrant  High x descendant  High x immigrant  *Sick leave* |  |  |  |  |
| Constant | -1.844***  (0.000) | -4.559***  (0.317) | 0.102  (0.344) | 0.892**  (0.365) |
| *N*  *R^2^* | 757,800  0.111 | 757,800  0.120 | 579,661  0.105 | 579,661  0.116 |
| *Note*. Reported standard errors (in parentheses) clustered on individuals.  Reference categories: low exposure and primary education.  Calendar year dummy variables included in regressions.  Signiﬁcance levels: ***.01; **.05; *.1 | | | | |

**Table 3. Results from GLS regression of exposure-reducing mobility, by sex**

**Table 4. Results from logistic regression of occupational mobility, by sex (OR)**

|  | Men | Women | |  |
| --- | --- | --- | --- | --- |
| *Job exposure* |  |  | |  |
| Low-middle | 1.038***  (0.007)  1.082***  (0.007)  0.904***  (0.006)  0.979***  (0.005)  1.201***  (0.009)  1.055***  (0.009)  1.010  (0.008)  0.985  (0.010)  1.067***  (0.011)  1.052***  (0.008)  0.935***  (0.009)  1.061***  (0.012)  1.060***  (0.008)  0.760***  (0.008)  0.945***  (0.011) | 1.043***  (0.007)  0.912***  (0.007)  1.083***  (0.010)  1.100***  (0.007)  1.184***  (0.008)  1.185***  (0.010)  1.155***  (0.010)  1.338***  (0.012)  1.106***  (0.012)  0.777***  (0.007)  1.455***  (0.014)  1.124***  (0.013)  0.919***  (0.011)  0.501***  (0.005)  0.591***  (0.008) | |  |
|  |  |  |  |  |
| High-middle |  |  |  |  |
|  |  |  |  |  |
| High |  |  |  |  |
|  |  |  |  |  |
| *Educ. level* |  |  |  |  |
| Secondary educ. |  |  |  |  |
| BA |  |  |  |  |
| MA/Ph.d. |  |  |  |  |
| *Job exposure x educ. level* |  |  |  |  |
| Low-middle x upper sec. |  |  |  |  |
| Low-middle x BA |  |  |  |  |
| Low-middle x MA/Ph.d. |  |  |  |  |
| High-middle x upper sec. |  |  |  |  |
| High-middle x BA |  |  |  |  |
| High-middle x MA/Ph.d. |  |  |  |  |
| High x upper sec. |  |  |  |  |
| High x BA |  |  |  |  |
| High x MA/Ph.d. |  |  |  |  |
| *N* | | 8,854,628 | 8,223,527 | |
| Pseudo R^2^ | | 0.023 | 0.045 | |
| *Note.* Odds ratios are reported, with standard errors (in parantheses) clustered on individuals  OR > 1: The exposure is associated with higher odds of the outcome  Reference categories: low exposure and primary education.  Calendar year dummy variables included in regressions.  Signiﬁcance levels: ***.01; **.05; *.1 | |  | |  |

|  | Men | Women | |  |
| --- | --- | --- | --- | --- |
| *Job exposure* |  |  | |  |
| High-middle | 3.241***  (0.453)  5.677***  (0.084)  1.130***  (0.014)  0.920***  (0.014)  0.660***  (0.011)  1.018  (0.017)  1.022  (0.201)  1.635***  (0.035)  1.084***  (0.019)  0.741*** (0.015)  1.678***  (0.039) | 2.268***  (0.034)  13.062***  (0.344)  1.071***  (0.012)  0.814***  (0.011)  0.713***  (0.011)  1.064***  (0.020)  0.735***  (0.014)  1.743***  (0.039)  1.008  (0.034)  0.236***  (0.007)  0.608***  (0.021) | |  |
|  |  |  |  |  |
| High |  |  |  |  |
|  |  |  |  |  |
| *Educ. level* |  |  |  |  |
| Secondary educ. |  |  |  |  |
| BA |  |  |  |  |
| MA/Ph.d. |  |  |  |  |
| *Job exposure x educ. level* |  |  |  |  |
| High-middle x upper sec. |  |  |  |  |
| High-middle x BA |  |  |  |  |
| High-middle x MA/Ph.d. |  |  |  |  |
| High x upper sec. |  |  |  |  |
| High x BA |  |  |  |  |
| High x MA/Ph.d. |  |  |  |  |
| *N* | | 757,800 | 579,661 | |
| Pseudo R^2^ | | 0.092 | 0.091 | |
| *Note.* Odds ratios are reported, with standard errors (in parantheses) clustered on individuals  OR > 1: The exposure is associated with higher odds of the outcome  Reference categories: low exposure and primary education.  Calendar year dummy variables included in regressions.  Signiﬁcance levels: ***.01; **.05; *.1 | |  | |  |

**Table 5. Results from logistic regression of downward occupational mobility, by sex (OR)**
